# Supplementary material for: The evolution of mechanisms to produce phenotypic heterogeneity in microorganisms
Source: Nat Commun. 2022 Jan 25;13:195. doi: 10.1038/s41467-021-27902-4 (PMC8789899; doi:10.1038/s41467-021-27902-4)
Supplement: Supplementary file 1 — Supplementary Information [file 41467_2021_27902_MOESM1_ESM.pdf]

The evolution of mechanisms to produce phenotypic heterogeneity in microorganisms: Supporting Information

Guy A. Cooper*[1,2], Ming Liu†[2], Jorge Peña‡[3], and Stuart A. West§[2]

[1]St. John's College, Oxford, United Kingdom
[2]Department of Zoology, University of Oxford, Oxford, United Kingdom
[3]Institute for Advanced Study in Toulouse, University of Toulouse Capitole, Toulouse, France

Overview

This supplementary information file is organized as follows. Supplementary Methods A re-examines key assumptions and approximations that simplified the analysis of the public goods model in the main text. Supplementary Methods B considers several models for alternative biological systems leading to different kinds of public goods. Both Supplementary Methods A and Supplementary Methods B demonstrate the robustness and generality of our results when relaxing our assumptions and approximations. Supplementary Methods C gives details on the simulation model that examines the possibility of intermediate coordination. Supplementary Discussion distinguishes different kinds of fitness costs that can be incurred by a mechanism to divide labour and discusses the potential role of functional constraints.

*E-mail address: guy.cooper@zoo.ox.ac.uk
†E-mail address: ming.liu@zoo.ox.ac.uk
‡E-mail address: jorge.pena@iast.fr
§E-mail address: stuart.west@zoo.ox.ac.uk

Supplementary Methods A Alternative modelling assumptions

Supplementary Methods A.1 Discrete proportions of helpers

In the main text, we approximated the optimal proportion of helpers in coordinated groups as a continuous variable $p^* \approx \hat{p} \in [0, 1]$. In reality, this quantity is discrete as it is given by $p^* = k^*/n$ and there can only be an integer number of helpers in the group (since $k^* \in \mathcal{N}$, then $p^* \in \mathcal{P}$).

Here, we show that this assumption is relatively innocuous. First, it is clear that p^* tends to $\hat{p}$ as n grows (Supplementary Figure 1A). Second, we find that even for relatively small group size n the predictions of the model are relatively independent of this assumption. To show this, we repeat our analysis without making the continuous approximation. That is, we evaluate $w_{\mathrm{C}}(p^*) > w_{\mathrm{R}}(p^*)$ where p^* is not approximated as $\hat{p}$ but is calculated numerically for each combination of parameters considered, so that $p^* \in \mathcal{P}$. The results of this analysis are shown in Supplementary Figure 1B. As expected, we find the broad results are similar to those of the continuous treatment, although we now find that in some cases division of labour is not favoured.

Supplementary Methods A.2 Optimal random specialisers

In the main text, we assumed that the probability of becoming a helper, q, was approximately equal to the optimal proportion of helpers (i.e., we let $q = \hat{p} \approx p^*$) (Supplementary Figure 1A). A more parsimonious assumption is that the probability of being a helper in random specialisers is the one optimising fitness, so that random specialisers play strategy $q = q^*$, with

$$q^* = \arg\max_{0 \leq q \leq 1} w_{\mathrm{R}}(q). \tag{S1}$$

This alternative modelling assumption makes the fitness of random specialisers larger (as $w_{\mathrm{R}}(q^*) \geq w_{\mathrm{R}}(p^*)$ necessarily holds) and thus will make, all else being equal, random specialisation more likely to be favoured over coordinated specialisation.

To find q^* for the linear public goods model, we take the derivative of Equation 17 in the main text with respect to q:

$$w'_{\mathrm{R}}(q) = (1 - c_R)\left(-b + h\frac{n-1}{n} - 2h\frac{n-1}{n}q\right). \tag{S2}$$

This derivative is decreasing in q (hence $w_{\mathrm{R}}(q)$ is concave) and has a single root q^* given by

$$q^* = \frac{h(n-1) - bn}{2h(n-1)} = \frac{1}{2}\left(1 - \frac{n}{n-1}\frac{b}{h}\right). \tag{S3}$$

Such root lies in the interval $(0, 1)$ if $w'_{\mathrm{R}}(0) = h(n-1)/n - b > 0$ holds, or equivalently, if $h/b > n/(n-1)$ holds, which we assume in the following. Expression (S3) is increasing in both n and h/b. We also note that

$$\hat{p} - q^* = \frac{b}{2h(n-1)} \geq 0, \tag{S4}$$

and hence (i) for essential cooperation ($b = 0$), $\hat{p} = q^*$, (ii) for non-essential cooperation ($b > 0$), $\hat{p}$ always overestimates q^*, but (iii) the difference between the two values is inversely proportional to n and goes to zero as n grows large. Thus, $\hat{p}$ approximates q^* relatively well for relatively large n, which justifies our use of $\hat{p}$ as an approximation in the main text (see Supplementary Figure 1A).

We further note that the derivative (S2) can be understood as a selection gradient on q. As such selection gradient is decreasing in q, the point q^* is not only a fitness maximum, but also the value to which evolution by small-step mutations would eventually lead, that is q^* is a convergence stable strategy [2, 6].

We present numerical results in Supplementary Figure 1C, evaluating the condition $w_{\mathrm{C}}(p^*) > w_{\mathrm{R}}(q^*)$ to determine when coordinated specialisation is favoured over random specialisation. Overall, we find similar qualitative results hold as in the simplified model.

Supplementary Methods A.3 Non-linear benefits to cooperation

In the main model, we assumed that reproductive fecundity depends linearly on the proportion of helpers in the group. Here, we consider the possibility that there is a non-linear dependence on the proportion of helpers by modelling reproductive fecundity after the sequence

$$f_{k,n} = b + h(k/n)^{\nu}, \tag{S5}$$

where $\nu > 0$ is a parameter controlling the shape of the return from an increasing proportion of helpers. If $\nu < 1$, there is a large initial return from the addition of the first helper, followed by a decelerating rate of return as the proportion of helpers increases (i.e., $f_{k,n}$ is concave; see Supplementary Figure 2A). Otherwise, if $\nu > 1$ there is a small initial return from the addition of the first helper followed by an accelerating rate of return as the proportion of helpers increases (i.e., $f_{k,n}$ is convex; see Supplementary Figure 2B). In all cases, (S5) is monotonically increasing from zero to one.

We considered the parameter discretisation, $n \in \{2, 4, \ldots, 38, 40\}$ and $h/b \in \{1, 1 + 9/24, \ldots, 1 + 23 \times 9/24, 1\}$ and, for each combination of parameters, we solved numerically for the optimal proportion of helpers, p^*, that maximises $G(p)$. For each combination of parameters, we then determined whether coordinated specialisation is favoured over random specialisation (i.e., whether $w_{\mathrm{C}}(p^*) > w_{\mathrm{R}}(p^*)$ holds), to ascertain whether accelerating ($\nu > 1$), or decelerating ($\nu < 1$) returns affect the predictions of our analysis. We find that the same broad results hold as in the linear public goods game (see Supplementary Figure 2C and 2D). We further find that accelerating benefits, in which there is a low initial return from helpers, can only lead to division of labour being favoured for a sufficiently high relative importance of cooperation (Supplementary Figure 2D).

Supplementary Methods B Alternative forms of cooperation

In the main model, we assumed that the fecundity of reproductives depends on the proportion of sterile helpers in the group, k/n. Here, we consider the conceptual underpinnings of this assumption (section Supplementary Methods B.1) and analyse alternative scenarios where reproductive fecundity depends either on the number of helpers in the group, k (section Supplementary Methods B.2), or on the ratio of helpers to reproductives, $k/(n-k)$ (section Supplementary Methods B.3). We also analyse a model in which helpers may also reproduce, albeit with diminished fecundity (section Supplementary Methods B.4). Further, our main model assumed that division of labour occurs only when groups reach their maximal size. Here we consider an extention of the model in which division of labour occurs in every generation of group growth (section Supplementary Methods B.5).

Supplementary Methods B.1 Reproductives benefit from the proportion of helpers

There are two key scenarios where reproductive fecundity can be modelled as depending on the proportion of helpers in the group, k/n.

First, the benefits provided by helpers may constitute a good that is consumed by its beneficiaries (sometimes termed a congestible or rivalrous good), wherein the benefit experienced by one individual proportionally decreases the amount of good that may benefit its neighbours [3, 5]. For instance, the secretion of an extracellular product that must be absorbed and digested in order to provide benefits (as happens in populations of *B. subtilis* and *A. cylindrica*) can be conceptualised as such a good [7, 12, 14]. In particular, if the good is non-excludible (i.e., all individuals use the benefits) then the benefits conferred to each reproductive is the total amount of help (in the linear case, approximated as the number of helpers, k) divided by the number of individuals in the group (n; reproductives and helpers alike) [5]. The fact that helpers also partake in the consumption of the public good may be considered a type of soaking [10].

Second, the amount of good that each helper provides may depend on the number of individuals in the group such that, all else being equal, a larger size entails less of a benefit to the group. For instance, helpers in *V. carteri* beat their flagella to keep the colony afloat at the optimal height in the water column for photosynthesis [9, 11]. In this case, the contribution of each helper (the degree to which it helps keep the colony at the right height) depends inversely on the size of the group as larger colonies are more difficult to keep afloat.

Supplementary Methods B.2 Reproductives benefit from the number of helpers

An alternative assumption is that the fecundity of reproductives depends on the number of helpers in the group, k. This would imply that the contribution of each helper to the total good does not depend on the size of the group and that the benefit conferred to one individual does not decrease the benefits available for another. The benefits provided by helpers in *S. enterica* infections is an example of such a good (a non-congestible or non-rivalrous good) [3, 5]. In this case, helpers trigger a host immune response that wipes out competing microbial strains so that reproductives may then proliferate without competition [1, 4]. Thus, the competitive advantage afforded by the host immune response is not used up or depleted by any of its beneficiaries, unless there are so few niches vacated that reproductives then compete with one another.

We would like to know whether we recover similar results as the ones we obtained assuming that reproductives benefit from the proportion of helpers in an alternative model in which the fecundity of reproductives depends on the number of helpers in the group, k. To this end we posit the following specific sequence for reproductive fecundity:

$$f_{k,n} = b + hk.$$

Replacing this expression into Equations 6 and 7, and by some abuse of notation (as now G depends explicitly on the group size n), we obtain

$$G(p) = (1-p)(b + hnp). \tag{S6}$$

Treating p as a continuous variable, we calculate the first derivative of G with respect to p as

$$G'(p) = hn - b - 2hnp.$$

This derivative is decreasing in p (i.e., $G(p)$ is concave), and has a single root $\hat{p}$ given by

$$\hat{p} = \frac{hn-b}{2hn} = \frac{1}{2}\left(1 - \frac{1}{n}\frac{b}{h}\right). \tag{S7}$$

Such root lies in the interval $(0,1)$ if $h/b > 1/n$, which we assume henceforth.

Using arguments similar to the ones we used in the Methods, it follows that we can approximate the fitness of a coordinated group as

$$w_{\mathrm{C}}(p^*) = (1 - c_{\mathrm{C}}) G(p^*) \approx (1 - c_{\mathrm{C}}) G(\hat{p}),$$

while the fitness of a randomly specialising group randomising with probability q can be written as

$$\begin{aligned} w_{\mathrm{R}}(q) &= (1 - c_{\mathrm{R}}) \sum_{k=0}^{n} \binom{n}{k} q^k (1-q)^{n-k} (1 - k/n)(b + hk) \\ &= (1 - c_{\mathrm{R}}) \left(b + hnq - bq - hnq^2 - hq(1-q)\right) \\ &= (1 - c_{\mathrm{R}}) \left(G(q) - hq(1-q)\right) \\ &= (1 - c_{\mathrm{R}}) \left(G(q) - hn\mathrm{Var}(Q)\right). \end{aligned}$$

Assuming that random specialisers play $q = p^*$, the condition for coordinated specialisation to be favoured over random specialisation is:

$$\mathrm{Var}(P^*) \frac{h}{G(p^*)} n > \underbrace{\frac{c_{\mathrm{C}} - c_{\mathrm{R}}}{1 - c_{\mathrm{R}}}}_{\gamma}. \tag{S8}$$

Further endorsing our approximation $p^* \approx \hat{p}$, we have

$$\mathrm{Var}(P^*) \approx \frac{(hn - b)(hn + b)}{4n^3 h^2}, \tag{S9}$$

$$\frac{h}{G(p^*)} \approx \frac{4h^2 n}{(hn + b)^2}, \tag{S10}$$

so that condition (S8) becomes

$$\frac{hn - b}{n(hn + b)} > \gamma. \tag{S11}$$

We present some numerical results in Supplementary Figure 3A evaluating the condition $w_{\mathrm{C}}(p^*) > w_{\mathrm{R}}(p^*)$. As in the main model where reproductives benefit from the proportion of helpers, we find that smaller group sizes (smaller n) and relatively more important cooperation (higher h/b) favour coordinated specialisation. We also find that less essential forms of cooperation ($h/b < 1$) may still favour division of labour.

Supplementary Methods B.3 Reproductives benefit from the ratio of helpers to reproductives

In a second alternative scenario, only reproductive individuals benefit from the collective good. In this case, the fecundity of reproductives is determined by the number of helpers (approximate amount of collective good) divided by the number of reproductives (the number of beneficiaries of the good). This gives the following expression for the fecundity of a reproductive in a group of size n with k helpers:

$$f_{k,n} = \left(b + h\frac{k}{n-k}\right)[k < n], \tag{S12}$$

where $[k < n]$ indicates that $f_{k,n} = 0$ if $k = n$.

Replacing this expression into Equations 7 and 6 of the main text and simplifying, we obtain

$$g_{k,n} = \left(b + \frac{(h-b)k}{n}\right)[k < n]. \tag{S13}$$

for the fitness of a group of size n with k helpers. This sequence is unimodal (first increasing, then decreasing) in k if $h > b$ holds, which we assume henceforth. In this case, $k^* = n - 1$ maximises $g_{k,n}$ for all n, and the optimal proportion of helpers is then $p^* = (n-1)/n$. It follows that the fitness for coordinated specialisers is given by

$$w_{\mathrm{C}}(p^*) = (1 - c_{\mathrm{C}}) g_{k^*,n} = (1 - c_{\mathrm{C}}) g_{n-1,n} = (1 - c_{\mathrm{C}}) \frac{b + (n-1)h}{n}. \tag{S14}$$

In contrast, the expected fitness for random specialisers can be calculated as

$$\begin{aligned} w_{\mathrm{R}}(q) &= (1-c_{\mathrm{R}})\sum_{k=0}^{n-1}\binom{n}{k}q^{k}\left(1-q\right)^{n-k}\left[b+(h-b)k/n\right] \\ &= (1-c_{\mathrm{R}})\left\{\sum_{k=0}^{n}\binom{n}{k}q^{k}\left(1-q\right)^{n-k}\left[b+(h-b)k/n\right]-\binom{n}{n}q^{n}\left(1-q\right)^{0}\left[b+(h-b)\right]\right\} \\ &= (1-c_{\mathrm{R}})\left(b+(h-b)q-hq^{n}\right). \end{aligned} \tag{S15}$$

At this point we deviate from our previous approximations and compare the fitness of coordinated specialisers and the fitness of random specialisers by evaluating the condition $w_{\mathrm{C}}(p^*) > w_{\mathrm{R}}(q^*)$, where q^* is the optimal probability of becoming a helper for random specialisers. We do this because this model results in a highly asymmetric group fitness $g_{k,n}$ for which the approximation $q^* \approx p^*$ is less accurate.

The derivative of $w_{\mathrm{R}}(q)$ (S15) with respect to q is given by

$$w'_{\mathrm{R}}(q) = (1-c_{\mathrm{R}})(h-b-hnq^{n-1}).$$

For $h > b$, this expression has a single root in the interval $(0, 1)$, given by

$$q^* = \left(\frac{h-b}{hn}\right)^{\frac{1}{n-1}}, \tag{S16}$$

which maximises $w_{\mathrm{R}}(q)$. Evaluating (S15) at $q = q^*$, and simplifying, we obtain the coordinated specialisation is favoured over random specialisation ($w_{\mathrm{C}}(p^*) > w_{\mathrm{R}}(q^*)$), when

$$\frac{b/n + h(n-1)/n}{b + (h-b)\left(\frac{h-b}{hn}\right)^{\frac{1}{n-1}} - h\left(\frac{h-b}{hn}\right)^{\frac{n}{n-1}}} > \frac{1-c_{\mathrm{R}}}{1-c_{\mathrm{C}}} \tag{S17}$$

holds.

Supplementary Figure 3B, graphically shows when coordinated specialisation is favoured over random specialisation. We find that that smaller group sizes (n) and relatively more important cooperation (higher h/b) favour coordinated specialisation.

In contrast to what we found when benefits depend on the number of helpers (cf. Supplementary Figure 3A), when the benefits depend on the ratio of helpers to reproductives (Supplementary Figure 3B) and the relative importance of cooperation (h/b) is high, larger group sizes (n) may still favour fully coordinated specialisation.

Supplementary Methods B.4 Reproductive division of labour with non-sterile helpers

Here, we consider the possibility that helpers also reproduce. For instance, this happens with the subset of *Bacilus subtilis* cells that produce protein degrading proteases (i.e., act as helper cells) but still produce offspring. Let $x \in [0, 1]$ be the degree to which helpers are specialised in the production of a public good. We assume that no specialisation ($x = 0$) entails no production of public goods and no personal fecundity cost, but that increasing specialisation (higher x) leads to a higher production of public goods at a linearly increasing personal cost to fecundity such that full specialisation ($x = 1$) means that helpers are sterile. At a given level of specialisation, the amount of public good produced by a helper is modelled as x^α, where $\alpha > 1$ is the scale of the efficiency benefit from specialisation. Combined, these assumptions give rise to

$$G(p) = p(1-x)(b + hx^\alpha p) + (1-p)(b + hx^\alpha p), \tag{S18}$$

where the first term is the per capita fitness of the group due to the fecundity of helpers, and the second term is the per capita fitness of the group due to the fecundity of reproductives. To find the fitness of coordinated specialisers, we assume that p is a continuous variable, and calculate the derivative

$$G'(p) = -2hx^{\alpha+1}p - xb + hx^\alpha,$$

which is decreasing in p (i.e., $G(p)$ is concave) and has a single root, $\hat{p}$, given by

$$\hat{p} = \frac{hx^{\alpha-1} - b}{2hx^\alpha} = \frac{1}{2}\left(x^{-1} - x^{-\alpha}\frac{b}{h}\right). \tag{S19}$$

Such a root lies in the interval $(0, 1)$ if and only if $hx^{\alpha-1} > b$ holds. Otherwise, the maximiser of $G(p)$ (and hence the optimal allocation of helpers) is given by $\hat{p} = 0$ (i.e., it is optimal to have no helpers). To make progress, we approximate the optimal allocation of helpers, p^*, by $\hat{p}$.

Note that the approximate optimal proportion (S19) (i) is independent of n; (ii) is an increasing function of h/b such that $\hat{p} = 1/(2x)$ when cooperation is essential ($b = 0$); and (iii) is an increasing function of the degree of helper specialisation x such that $\hat{p} = (h - b)/(2h)$ when helpers are sterile ($x = 1$; this recovers Equation (S7) as a particular case).

On the one hand, we obtain an approximation to the fitness of fully coordinated specialisers by letting $p^* \approx \hat{p}$, which leads to

$$w_{\mathrm{C}}(p^*) = (1 - c_{\mathrm{C}})G(p^*) \approx (1 - c_{\mathrm{C}})G(\hat{p}). \tag{S20}$$

On the other hand, the fitness of random specialisers can be calculated as

$$\begin{aligned}
w_{\mathrm{R}}(q) &= (1 - c_{\mathrm{R}}) \sum_{k=0}^{n} \binom{n}{k} q^k (1 - q)^{n-k} \left[(k/n)(1 - x)(b + hx^{\alpha}k/n) + (1 - k/n)(b + hx^{\alpha}k/n)\right] \\
&= (1 - c_{\mathrm{R}}) \left\{b(1 - x)\mathrm{E}\left[K/n\right] + hx^{\alpha}(1 - x)\mathrm{E}[K^2/n^2] + b\mathrm{E}\left[(1 - K/n)\right] + hx^{\alpha}\mathrm{E}[K/n - K^2/n^2]\right\} \\
&= (1 - c_{\mathrm{R}}) \left\{b(1 - x)q + hx^{\alpha}(1 - x)\frac{nq(1 - q) + n^2q^2}{n^2} + b(1 - q) + hx^{\alpha}\left[q - \frac{nq(1 - q) + n^2q^2}{n^2}\right]\right\} \\
&= (1 - c_{\mathrm{R}}) \left\{b(1 - x)q + hx^{\alpha}(1 - x)\left[\frac{q(1 - q)}{n} + q^2\right] + b(1 - q) + hx^{\alpha}\left[q(1 - q) - \frac{q(1 - q)}{n}\right]\right\} \\
&= (1 - c_{\mathrm{R}}) \left\{q(1 - x)\left(b + hx^{\alpha}q\right) + hx^{\alpha}(1 - x)\frac{q(1 - q)}{n} + (1 - q)\left(b + hx^{\alpha}q\right) - hx^{\alpha}\frac{q(1 - q)}{n}\right\} \\
&= (1 - c_{\mathrm{R}}) \left\{G(q) - hx^{\alpha+1}\frac{q(1 - q)}{n}\right\} \qquad \text{(S21)} \\
&= (1 - c_{\mathrm{R}}) \left(G(q) - hx^{\alpha+1}\mathrm{Var}(Q)\right), \qquad \text{(S22)}
\end{aligned}$$

where we have made use of the first two moments of the binomial distribution, $\mathrm{E}[K] = nq$, $\mathrm{E}[K^2] = nq(1 - q) + (nq)^2$, of expression (S18), and of the fact that $\mathrm{Var}(Q) = q(1 - q)/n$.

Assuming that random specialisers play the strategy $q = p^*$, their fitness can be approximated by

$$w_{\mathrm{R}}(p^*) = (1 - c_{\mathrm{R}})\left(G(p^*) - hx^{\alpha+1}\mathrm{Var}(P^*)\right), \tag{S23}$$

where $P^* = K^*/n$ and $K^* \sim \mathrm{Binomial}(n, p^*)$ (and hence $\mathrm{Var}(P^*) = p^*(1 - p^*)/n$). Comparing expressions (S20) and (S22), it follows that coordinated specialisation is favoured over random specialisation (i.e., $w_{\mathrm{C}}(p^*) > w_{\mathrm{R}}(p^*)$ holds) if

$$\mathrm{Var}(P^*)\frac{hx^{\alpha+1}}{G(p^*)} > \gamma. \tag{S24}$$

Again, condition (S24) makes it explicit that the fecundity benefit of coordination over random specialisation can be decomposed into a measure of the deviation from the optimal allocation of labour, $\mathrm{Var}(P^*)$, and a quantity that captures the relative cost of deviating from the optimal proportion of helpers, $hx^{\alpha+1}/G(p^*)$.

To obtain a simple expression of condition (S24) in terms of our parameters n, b and h, we approximate p^* by $\hat{p}$ as given in (S19). Substituting this approximation, condition (S24) becomes:

$$\frac{(hx^{\alpha-1} - b)(hx^{\alpha-1}(2x - 1) + b)}{n(hx^{\alpha-1} + b)^2} > \gamma. \tag{S25}$$

The left-hand side of equation S25 is decreasing in the size of the group n. Numerical examination also reveals that the left-hand side is increasing in the degree of helper specialisation, x, and that it is unimodal in the relative benefits of cooperation, h/b, and in the efficiency benefits of specialisation, α (increasing and then decreasing). We show these results graphically in Supplementary Figure 3C and 3D for two different values of helper specialisation.

When helpers are less specialised (smaller x), more essential cooperation (h/b) can lead to coordinated specialisation being less likely to evolve (Supplementary Figure 3C). This occurs because more essential cooperation leads to a higher proportion of helpers ($\hat{p}$), which can increase to values greater than $1/2$ when helpers are not sterile ($x < 1$) (Equation S19). When a high proportion of non-sterile helpers is favoured ($\hat{p} \approx 1$), the expected variance in the proportion of helpers can become very small ($\mathrm{Var}(P^*) \approx 0$) and thus random groups are much less likely to deviate from the optimal proportion of helpers.

Supplementary Methods B.5 Reproductive division of labour as groups grow

We outline here a simulation model in which cells divide labour in every generation of the group-growth cycle. We simulate group-growth cycles using either random specialisation or coordinated specialisation in order to estimate the average fitness of each mechanism to divide labour in different scenarios. We assume that the social interactions are given by a linear public goods game as the one presented in the main text. For simplicity, although we investigate division of labours in groups of finite size, we implicitly assume that the size of groups is large. In particular, we make the approximation $p^* \approx \hat{p}$, with $\hat{p}$ given by Equation 14 of the main text.

For each simulation, we assume that groups start with one reproductive and that one cell is added to the group each generation of the group-growth cycle until the group has reached a total of n cells. Let $t \in T = \{1, 2, \ldots, n\}$ index the generation of the group growth such that in generation t, there are t cells in the group. Let p_t be the proportion of helpers in the group at generation t. Since groups start with one reproductive, $p_1 = 0$.

For groups that specialise randomly with helper probability q, we assume that each generation of the group growth cycle the new cell adopts a helper phenotype with probability q and otherwise is a reproductive. Thus, at generation $t > 1$, we have $p_t = (p_{t-1}(t-1)+1)/t$ with probability q and $p_t = (p_{t-1}(t-1))/t$ with probability $1-q$. The proportion of helpers in the final group of the life cycle, p_n, can take a value in the set $\{0, 1/n, \ldots, (n-1)/n\}$.

For groups that specialise with coordination, we assume that new cells adopt the phenotype that minimises the absolute difference between the proportion of helpers in the group and the optimal proportion of helpers, $p^* \approx \hat{p} = (h-b)/(2h)$ (cf. Equation 14 of the main text). Hence, in generation $t > 1$, if $|(p_{t-1}(t-1)+1)/t - p^*| > |p_{t-1}(t-1))/t - p^*|$ then the new cell adopts a reproductive phenotype, and $p_t = (p_{t-1}(t-1))/t$. On the other hand if $|(p_{t-1}(t-1)+1)/t - p^*| < |p_{t-1}(t-1)/t - p^*|$, then the new cell adopts a helper phenotype, and $p_t = (p_{t-1}(t-1)+1)/t$.

For each combination of parameter values considered, we simulated a large number of group-growth cycles (500 replications) for each mechanism in order to estimate the average fitness of each mechanism (w_{C} and w_{R}). In each simulation, the fitness of the group at the end of the life cycle was calculated as the product of the group fecundities at each generation,

$$\prod_{t \in T} (1-c)(1-p_t)(b + hp_t), \tag{S26}$$

where $(1-p_t)(b+hp_t)$ is the average fecundity of the group in generation t (cf. Equation 12 of the main text) and where we set $c = c_{\mathrm{C}}$ for coordinated groups, and $c = c_{\mathrm{R}}$ for randomly specialising groups. Once again, we assume that the probability that random specialisers adopt a helper role is equal to the optimal proportion of helpers, $q = p^* \approx \hat{p}$. We also assumed that the cost of the mechanisms c_{C} and c_{R} is independent of group size, n, for both mechanisms.

Supplementary Figure 3E and 3F illustrate our results. We can see how smaller final group sizes (n), more essential cooperation (higher h/b), and lower relative costs of coordination (γ) favour coordinated specialisation.

Supplementary Methods C Evolution of the level of coordination (individual based simulations)

In the first analysis of the main text, we assumed that all individuals in a coordinated group interact with one another and so have complete information about the phenotypes of their social partners when specialising. Consequently, coordinated groups are fully coordinated and always contain the optimal proportion of helpers to reproductives, p^*. Here we relax this assumption and use individual based simulations to determine the optimal level of coordination.

We provide a heuristic model where increasing the number of cell-to-cell interactions within the group can lead incrementally to a more precise allocation of labour. We consider a costly trait $s \in [0,1]$ (the level of coordination) that is equal to the independent probability that any two cells in the group interact with each other. When a focal individual specialises, it adopts a helper role depending on how the proportion of helpers amongst cells that it interacts with compares to a critical threshold, $t \in [0,1]$. Variables s and t are co-evolving traits in our simulations; they influence both the degree to which groups are coordinated and the degree to which labour is divided (i.e., the realised proportion of helpers, p). As the expected connectivity of the group increases (i.e., higher s, more one-to-one interactions), there is a higher cost of coordination paid by the group. However, this higher cost of coordination may be offset by the increased amount of information afforded to each individual when specialising and thus an increased chance that the group ends up with the optimal proportion of helpers, p^*. This trade-off means that in some cases the optimal strength of coordination may in fact be intermediate ($0 < s < 1$), rather than the full coordination considered in the main analysis ($s = 1$).

In the following, we outline our individual-based model. First, we specify how the simulations are initialised (section Supplementary Methods C.1). Second, given a group with a particular level of coordination (s), we specify how we determine which individuals interact with one another (section Supplementary Methods C.2). Third, given a group with an interaction network and target proportion of helpers (t), we describe how the allocation of labour within the group is determined (sec Supplementary Methods C.3). Fourth, given the allocation of labour, we specify how the fitness of each group is determined (section Supplementary Methods C.4). Finally, we describe how the individuals in the population compete globally from one generation to the next, and how mutation may affect the trait values of s and t (section Supplementary Methods C.5). Results for the evolved level of coordination and target proportion of helpers (section Supplementary Methods C.6) are shown in Supplementary Figure 4.

Supplementary Methods C.1 Simulation initialisation

We create a population of approximately N_{T} cells, sorted into groups of n cells. Specifically, we set the number of groups in the population as $N_{\mathrm{g}} = \lceil N_{\mathrm{T}}/n \rceil$, where $\lceil a \rceil$ is the least integer larger than or equal to a. Thus, the population size, $N_{\mathrm{g}}n$, is relatively constant while the number of groups increases as the group size decreases. This reduces the effect of demographic stochasticity across simulations. All individuals in the population are characterised by their trait values s and t. We assume that all groups are founded by a single asexual individual, and thus that groups are clonal and that all individuals in the same group have the same trait values. At the beginning of the simulation, we assume that all individuals in the population have no propensity for either division of labour or coordination (i.e., $s = 0$, $t = 0$).

Supplementary Methods C.2 Coordination network

Given a group with a particular level of coordination, s, we determine the between-cell coordination network by constructing a random graph $\mathcal{G}(n, s)$ where the n nodes correspond to the n cells in the group and where each possible edge (representing the interaction between a given pair of cells) occurs independently with probability s [8]. Thus, for a given s, the expected number of interacting pairs is $sn(n-1)/2$. If $s = 0$, there are no interacting pairs and individuals do not know the phenotypes of any other group members. If $s = 1$, all individuals interact with all other individuals in the group and know whether they are helpers or reproductives. In between these two extremes, individuals may only interact with a subset of the cells in the group and know only partial information about the proportion of helpers in the group.

Supplementary Methods C.3 Individual specialisation

Before we can describe how individuals within the group specialise in this framework, we need to establish a few definitions. For a given interaction network, $\mathcal{G}$, let $\mathcal{V} = \{v_1, \dots, v_n\}$ be the set of nodes (individuals or cells) and $\mathcal{E}$ be the set of edges, such that $e_{ij} \in \mathcal{E}$ if individuals v_i and v_j interact. We define an *allocation of labour*

as the map $a : \mathcal{V} \to \{0, 1\}$ such that $a(v_i) = 1$ if individual v_i is a helper and $a(v_i) = 0$ if v_i is a reproductive. The realised proportion of helpers to reproductives in the group is then given by $p = \sum_{i=1}^{n} a(v_i)/n$. We denote by $\mathcal{N}_\mathcal{G}(v_i)$ the open neighbourhood of individual v_i. The degree of v_i, d_i, is the number of cells in its open neighborhood, that is, $d_i = |\mathcal{N}_\mathcal{G}(v_i)|$. Using these definitions we can write the *open proportion of helpers in the neighbourhood* of individual v_i as $p_{(i)} = \sum_{v_j \in \mathcal{N}_\mathcal{G}(v_i)} a(v_j)/d_i$, that is, the fraction of its neighbours that are helpers. If an individual has no neighbours ($d_i = 0$), then the open proportion of helpers is not defined and the allocation of labour proceeds differently.

For a particular group (given a set of cells $\mathcal{V}$ and an interaction network $\mathcal{E}$), we determine the allocation of labour, a, using a threshold model. First, we assume that the group begins with an initial allocation of all reproductives ($a(v) = 0, \forall v \in \mathcal{V}$). This implicitly presupposes that reproduction is the default phenotype of cells. Then, for $\tau \geq n$ time steps, we randomly sample with replacement one cell $v_i \in \mathcal{V}$ at a time from the group. We sample with replacement and set $\tau \geq n$ so that a cell that has "committed" to a particular phenotype may still switch if too many of its neighbours have adopted the same choice. This emphasises that the allocation of labour process is dynamic and that developmental trajectories are plastic/responsive to their social environment. Each chosen cell, v_i, considers the open proportion of cells in its neighbourhood $p_{(i)}$ and chooses to adopt a helper or reproductive phenotype depending on its target proportion of helpers, t. If $p_{(i)} < t$, then the cell adopts a helper phenotype ($a(v_i) = 1$) as there are fewer helpers in its neighbourhood than its target proportion of helpers. If $p_{(i)} > t$, then the cell adopts a reproductive phenotype ($a(v_i) = 0$) as there are more helpers in its neighbourhood than its target proportion of helpers. If the open proportion of helpers is equal to the target proportion of helpers ($p_{(i)} = t$), then the cell adopts either phenotype with equal probability. If the individual has no neighbours ($d_i = 0$), then the individual behaves as a random specialist with helper probability equal to the target proportion of helpers ($q = t$).

Supplementary Methods C.4 Fitness

Once labour is allocated within the group, we calculate the fitness of the group using the fecundity equation of the linear public goods model (Equation 12 of the main text) with $b = 1 - \epsilon$ and $h = \epsilon$, where ϵ is a measure of how essential cooperation is. We assume that the cost of coordination is given by $c_\mathrm{C} = c(1 - e^{-\chi sn(n-1)/2})$, where $sn(n-1)/2$ is the expected number of cell-to-cell interactions, $c \in [0, 1]$ controls the scale of the cost, and $\chi > 0$ determines how diminishing the cost is. We assume that the cost of coordination depends directly on the expected number $sn(n-1)/2$ of cell-to-cell interactions rather than the realised number of cell-to-cell interactions because the former captures the effort that each individual puts into coordination instead of how successful its efforts actually were, and so is under evolutionary control.

Supplementary Methods C.5 Creating a new generation

Each generation, we create the new population by sampling group founders from individuals of the previous generation. That is, we randomly pick individuals with a probability proportional to the relative fitness of each group. The process repeats N_g times with replacement. Each sampled individual forms a group of n individuals with inherited trait values s and t. When the founder individual is sampled, we assume that there is a chance μ of having a mutation in one of the traits. If a mutation happens, the trait value is perturbed by adding a normally-distributed random number, $\delta \sim \mathrm{Normal}(0, \sigma^2)$, where σ^2 is the variance in the size of the mutation, truncated between zero and one.

Supplementary Methods C.6 Simulation results

We performed simulations for each combination of group size $n \in \{2, 4, \ldots, 38, 40\}$ and essentiality of cooperation $\epsilon \in \{1/2, 1/2 + 1/38, \ldots, 1/2 + 18/38, 1\}$. For each simulation we determined the number of groups as $N_\mathrm{g} = \lceil N_\mathrm{T}/n \rceil$, where $N_\mathrm{T} = 10,000$. All individuals are initialised with no coordination or no propensity for division of labour (i.e., $s = t = 0$). Next, we proceeded in each generation by determining the interaction network of each group (given its strength of coordination, s), and then each group's allocation of labour (given its target proportion of helpers, t). We then calculated the fitness of each group and sampled individuals for the next generation with a probability proportional to fitness. We assumed that mutations occur with probability $\mu = 0.001$ and that the expected variance in the mutation size was $\sigma^2 = 0.1$. We set $c = 0.1$ and $\chi = 0.01$ (parameters of the cost of coordination).

The results of the simulations are shown Supplementary Figure 4. We examined 400 combinations of group size and essentiality and ran the simulation 10 times for each parameter combination. Each simulation lasted 30,000 generations. For each simulation, we denote by $\hat{s}$ and $\hat{t}$ the average trait values across the population

for a given generation. We denote by $\bar{s}$ and $\bar{t}$ the across-simulation average of $\hat{s}$ and $\hat{t}$ for each generation. The evolutionary outcomes, s^* and t^*, are estimated as the average $\bar{s}$ and $\bar{t}$ in the last 3,000 generations of the simulations. We show the evolutionary outcome s^* as the main results but also plot 10 individual time series of $\hat{s}$ and $\hat{t}$ and the across-simulation average $\bar{s}$ and $\bar{t}$. To account for simulation stochasticity, we categorised random specialisation as any strategy for which $s^* < 0.1$, fully coordinated specialisation as any strategy for which $s^* > 0.9$ and intermediate specialisation as any strategy for which $0.1 < s^* < 0.9$. In Supplementary Figure 4, we show the results for s^* and t^* in these simulations, finding that smaller group sizes (n) and higher essentiality of cooperation (ϵ) favours higher levels of coordination (s^*) and that more essential cooperation (higher ϵ) favours a higher proportion of helpers (t^*). This is in agreement with the results of the previous analyses, while also highlighting that the level of coordination can also be a factor shaped by natural selection.

Supplementary Discussion   Costs, benefits, and functional constraints

When considering the relative costs and benefits of random versus coordinated specialisation, it is useful to keep in mind some key distinctions.

There are two key categories of costs that groups may pay. Firstly, a group pays what we term a “metabolic” cost from its mechanism to divide labour. This is a fixed cost that occurs at the time of differentiation into a helper or a reproductive role. It can arise from the resource cost of the molecular machinery required to coordinate or from the feedback mechanism that allows for random specialisation. This cost can also arise from the reproduction delay that may be required for between-cell signalling and coordination. The more complex the machinery required to coordinate phenotypes or the longer it takes to coordinate, the larger the metabolic cost of coordination will be.

Secondly, there is a relative cost paid when groups deviate from the group phenotype with the highest fitness value. Generally, this may be termed the “stochastic” or “functional cost” of the mechanism. In our main model, this cost is only paid by groups composed of random specialisers, which may deviate from the optimal proportion of helpers in the group. In the simulation model with a simple spatial structure, a functional cost can arise from an uneven distribution of helpers. When division of labour is only effective with a precise group composition, then the functional cost of deviation will be high.

When evaluating the different costs of each mechanism, it is useful to distinguish between two different time periods in the group life cycle. Cells differentiate into helpers or reproductives during the first time period. Helpers then provide benefits to reproductives during the second time period. Metabolic costs arise during the first time period, whereas functional costs are relevant to the second time period. For instance, all *Salmonella enterica* cells are located in the gut lumen during the first time period, in which some cells differentiate into helpers [1]. The second time period begins after a time gap, at which point helpers have migrated to the gut tissue to trigger a host immune response while reproductives remain safely in the gut lumen.

This highlights that functional costs depend on the group composition when cooperation occurs and not when cells are differentiating. For instance, in *Dyctiostelium discoideum*, pre-stalk cells are initially distributed seemingly at random across the cell-mass [13]. Subsequently, differential migration (behaviour) of pre-stalk and pre-spore cells leads to the formation of the fruiting body in the second time period. Whether the mechanism produces a viable group phenotype (low functional cost) depends on the arrangement of cells at the end of cell migration and not on the initial spatial distribution of cells in the cell mass. In particular, a group could have random specialisation (to cell types), and then coordinated or uncoordinated migration to positions appropriate for each cell type.

Finally, the canonical examples of division of labour in microorganisms are the end points of an evolutionary process, wherein group phenotypes such as those of *D. discoideum* and *Volvox carteri* have been refined into intricate forms [11, 13]. When division of labour initially arose, group phenotypes will have been less elaborate and thus the potential metabolic and functional costs of each mechanism could have been much reduced in comparison to their modern forms.

Supplementary Figures

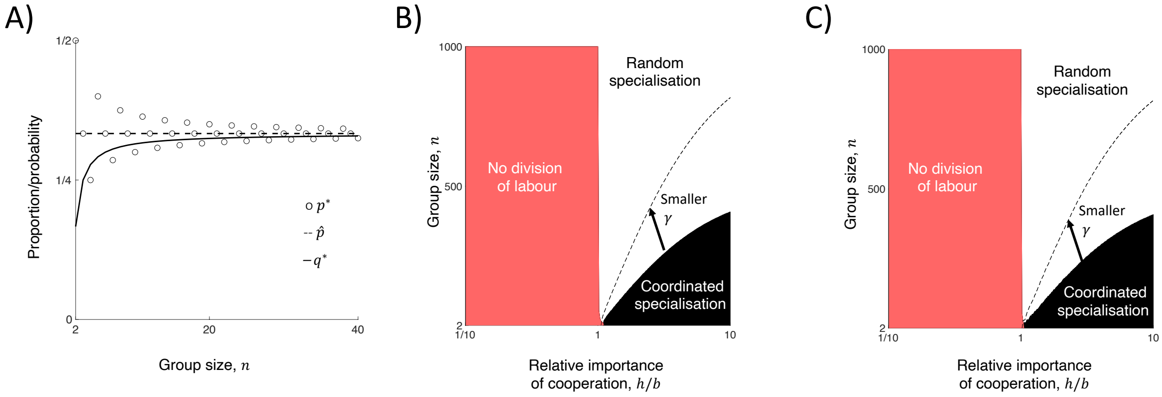

Supplementary Figure 1: **Alternative modelling assumptions**. A) Optimal proportion of helpers (p^*; *open circles*), its continuous approximation ($\hat{p}$; *dashed line*), and the probability optimising fitness for random specialisers (q^*; *solid line*) as a function of group size n. In the model presented in the main text, we assumed that the probability that random specialisers adopt a helper role was equal to the optimal proportion of helpers in the group (p^*) as opposed to the probability that optimises fitness for random specialisers (q^*). In turn, we approximated this optimal proportion of helpers by a continuous variable $\hat{p}$. These three quantities converge to the same value as group size (n) increases. Here, $b = 1/4$ and $h = 3/4$, leading to $\hat{p} = (1/2)(1 - b/h) = 1/3$. B) Favoured mechanism (*coordinated specialisation* if $w_{\mathrm{C}}(p^*) > w_{\mathrm{R}}(p^*)$; *random specialisation* if $w_{\mathrm{C}}(p^*) < w_{\mathrm{R}}(p^*)$) without approximating p^* by $\hat{p}$ (as in the main text). Instead, we evaluate the optimal value p^* numerically for each parameter combination considered. Division of labour is not favoured (i.e., the optimal proportion of helpers is zero) in the *red* region. C) Favoured mechanism (*coordinated specialisation* if $w_{\mathrm{C}}(p^*) > w_{\mathrm{R}}(p^*)$; *random specialisation* if $w_{\mathrm{C}}(p^*) < w_{\mathrm{R}}(p^*)$), while allowing for the probability that random specialisers adopt a helper role to evolve to the value that maximises the fitness of random specialisers (q^*). In both scenarios B) and C), we find that smaller group sizes (smaller n), relatively more important cooperation (higher h/b), and smaller relative metabolic costs of coordination (smaller γ) favour coordinated specialisation, in agreement with the results presented in the main text. In both cases, the relative importance of cooperation, h/b, is plotted on a log scale. In both cases we set γ to either 1×10^{-3} (smaller relative cost of coordination) or 2×10^{-3} (larger relative cost of coordination).

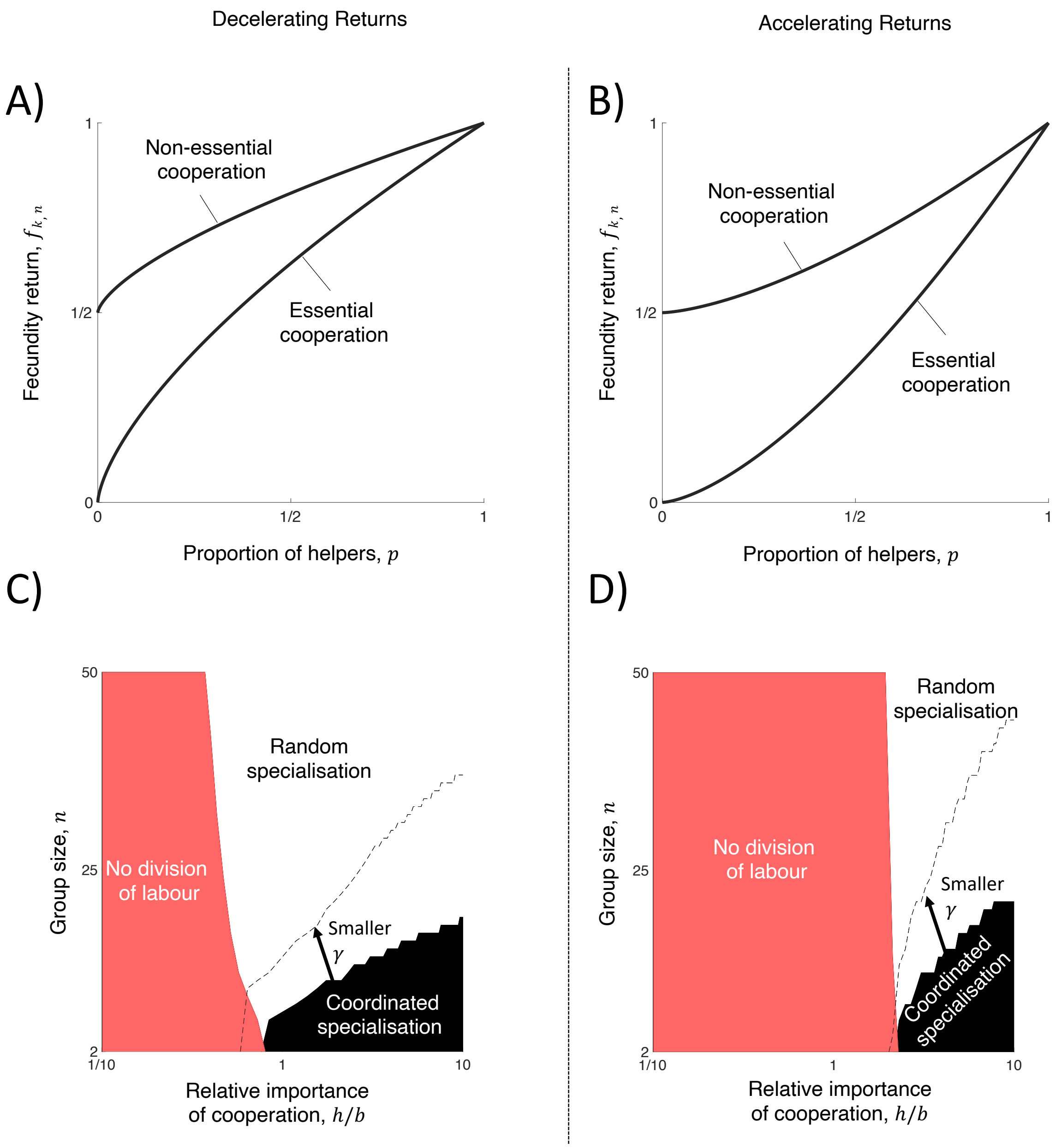

Supplementary Figure 2: **Non-linear public goods**. We consider an extension of the model allowing the fecundity of reproductives to depend non-linearly on the proportion of helpers in the group. (A) If $\nu < 1$, then the presence of helpers provides an initially large increase in the fecundity of reproductives but then only provides benefits at a decreasing rate as the proportion of helpers goes up (decelerating cooperation; here $\nu = 2/3$). (B) If $\nu > 1$, then the presence of helpers provides no initial increase in the fecundity of reproductives but then provides benefits at an increasing rate as the proportion of helpers goes up (accelerating cooperation; here $\nu = 3/2$). In both A) and B) we have set $b = h = 1/2$ for non-essential cooperation and $b = 0$ and $h = 1$ for essential cooperation. (C and D) Favoured mechanism (*coordinated specialisation* if $w_C(p^*) > w_R(p^*)$; *random specialisation* if $w_C(p^*) < w_R(p^*)$) with fecundity sequence given by Eq. (S5) and either $\nu = 3/4$ (decelerating cooperation; panel C) or $\nu = 4/3$ (accelerating cooperation; panel D). In both scenarios, we find that smaller group sizes (lower n), relatively more important cooperation (higher h/b) and a smaller relative metabolic cost of coordination (lower γ) favour coordinated specialisation. In both cases, the relative importance of cooperation, h/b, is plotted on a log scale. We set γ either to $= 0.02$ (lower relative cost of coordination) or 0.04 (higher relative cost of coordination). The *red* region shows where division of labour is not favoured, that is, where the optimal proportion of helpers is zero.

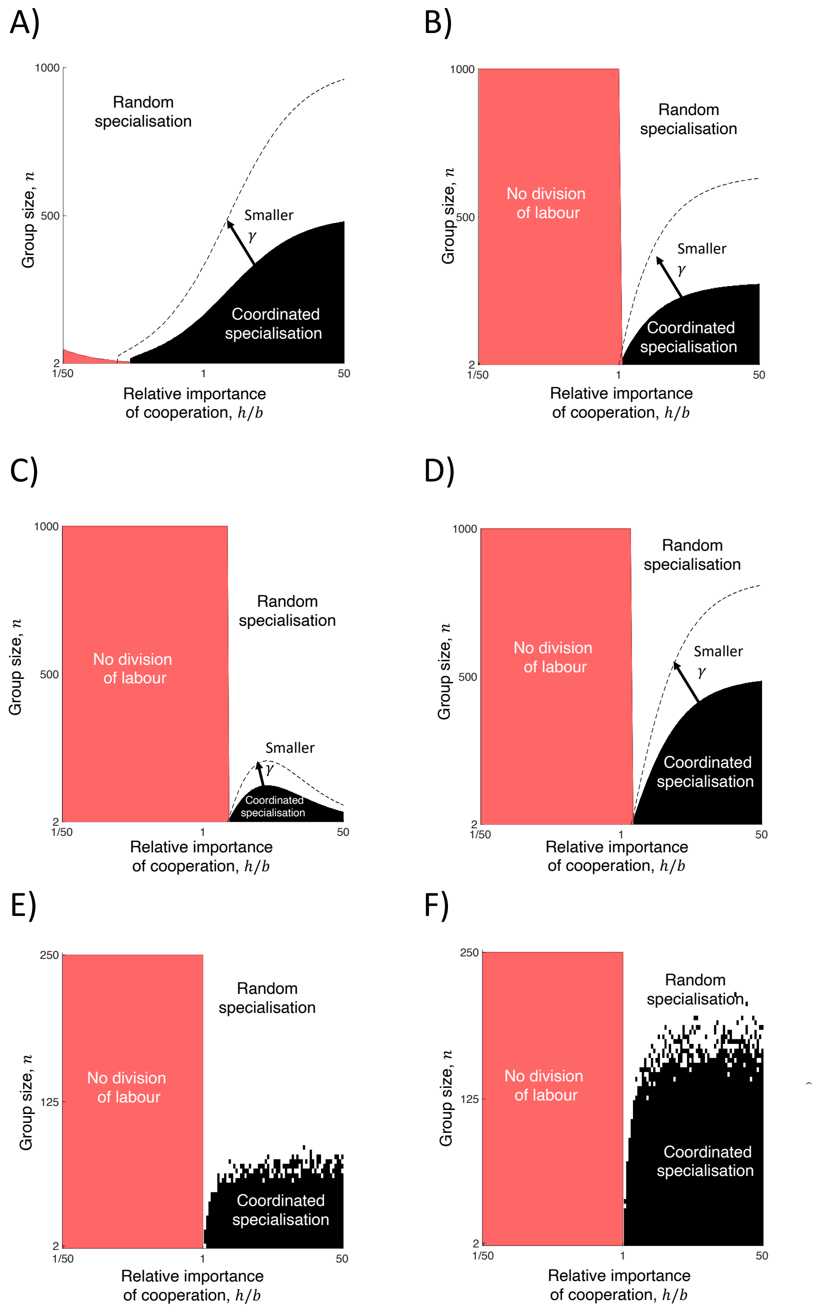

Supplementary Figure 3: **Alternative biological scenarios**. We consider the relative fitness advantage of coordination over random specialisation as a function of the final size of the group n and the relative importance of cooperation, h/b, in different biological scenarios. (A) Fecundity of reproductives depends on the number of helpers in the group. $\gamma = 1 \times 10^{-3}$ (lower relative costs of coordination) or $\gamma = 2 \times 10^{-3}$ (higher relative cost of coordination). (B) Fecundity of reproductives depends on the ratio of helpers to reproductives. $\gamma = 1 \times 10^{-2}$ (lower relative costs of coordination) or $\gamma = 2 \times 10^{-2}$ (higher relative cost of coordination). (C and D) Helpers are non-sterile but less specialised helpers spend a proportion x of their resources on producing the public good (panel C: $x = 1/2$; panel D: $x = 3/4$). In both cases smaller group sizes (n), relatively more important cooperation (higher h/b) and smaller relative costs of coordination (γ) favour coordinated specialisation. More specialised helpers (higher x) favours more coordinated specialisation. In both cases, $\alpha = 2$. For both panels C and D: $\gamma = 6 \times 10^{-4}$ (lower relative costs of coordination) or $\gamma = 1 \times 10^{-3}$ (higher relative cost of coordination). (E and F) Division of labour occurs in every generation as the group grows (panel E: $\gamma = 0.03$; panel F: $\gamma = 0.015$). Smaller group sizes (n), relatively more important cooperation (higher h/b) and smaller relative costs of coordination (γ) favour coordinated specialisation. In all cases, the relative importance of cooperation, h/b, is plotted on a log scale. In the *red* region division of labour is not favoured, that is, the optimal proportion of helpers is zero.

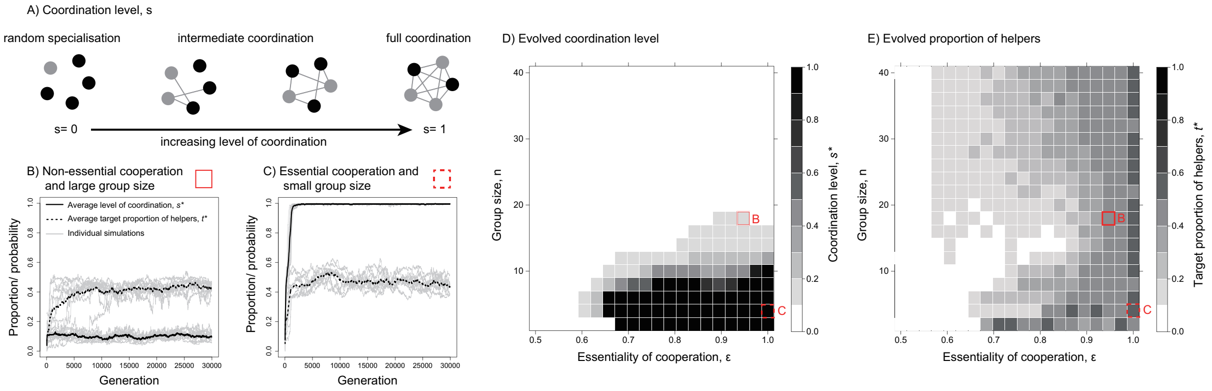

Supplementary Figure 4: **Evolution of the level of coordination**. (A) If the level of coordination may evolve, then a spectrum of possible mechanisms could arise. At one extreme ($s = 0$), individuals may not coordinate at all (random specialisation). At the other extreme ($s = 1$), all individuals coordinate with one another and the variance in the realised proportion of helpers is minimised (fully coordinated specialisation). In between ($0 < s < 1$), intermediate forms of coordination may arise in which not all individuals coordinate with one another and sub-optimal proportions of helpers can occur. (B and C) We present the across-simulation average level of coordination ($\bar{s}$, *solid line*) and target proportion of helpers ($\bar{t}$, *dashed line*) that evolves. We also plot individual simulation time series for the trait values (*gray lines*), where each line corresponds to a population average. We find that the traits converge stably within hundreds of generations. Moreover, the evolutionary outcomes (estimated by s^* and t^*), depend on demographic and environmental conditions (panel B corresponds to the *red box, solid line* in panel D, whereas panel C corresponds to the *red box, dashed line* in panel D). Separate simulations show that these results are independent of starting conditions. (D) Smaller groups (lower n) and more essential cooperation (higher ϵ) favour the evolution of fully coordinated specialisation ($s^* \approx 1$). Intermediate coordination ($0 < s^* < 1$) may evolve in more moderate conditions. (E) Higher essentiality of cooperation (higher ϵ) leads to a higher proportion of helpers. Group size (n) has less of an effect on the target proportion of helpers. $c = 0.1$ and $\chi = 0.01$.

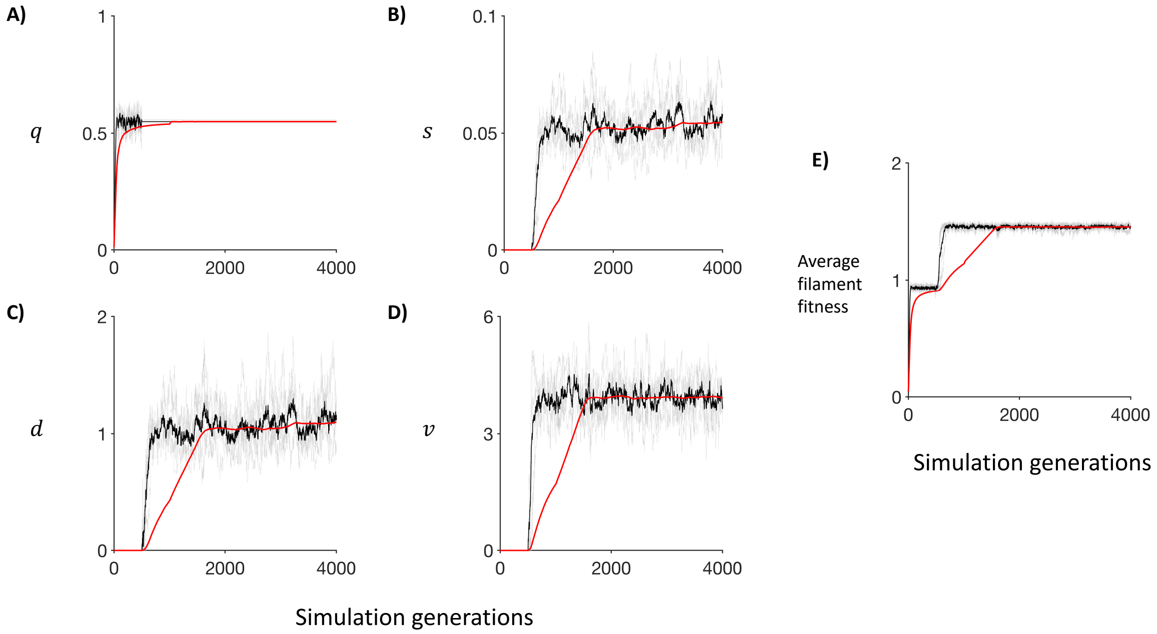

Supplementary Figure 5: **Convergence to optimal trait values (cyanobacteria model)**. We show the convergence of trait values and average filament fitness across 5 independent simulations for the case of essential cooperation ($\phi = 0$) and very local cooperation ($\eta = 0.1$). A) Convergence of the helper probability, q. B) Convergence of the signal production, s. C) Convergence of the signal threshold, d. D) Convergence of the signal response sensitivity, v. E) Convergence of average filament fitness. In each case, the grey lines show the trait and fitness values for each of the 5 independent simulations, the black line shows the average trait value or fitness across the 5 independent simulations for that particular generation, and the red line shows the rolling average over the last 1000 generations (truncated if simulation generation is less than 1000). Combined, these show that the rolling averages are stable by the end of the simulations, and that the trait values have approximately converged to the final rolling average within 100-200 generations of being allowed to evolve. The convergence of average filament fitness is particularly stable.

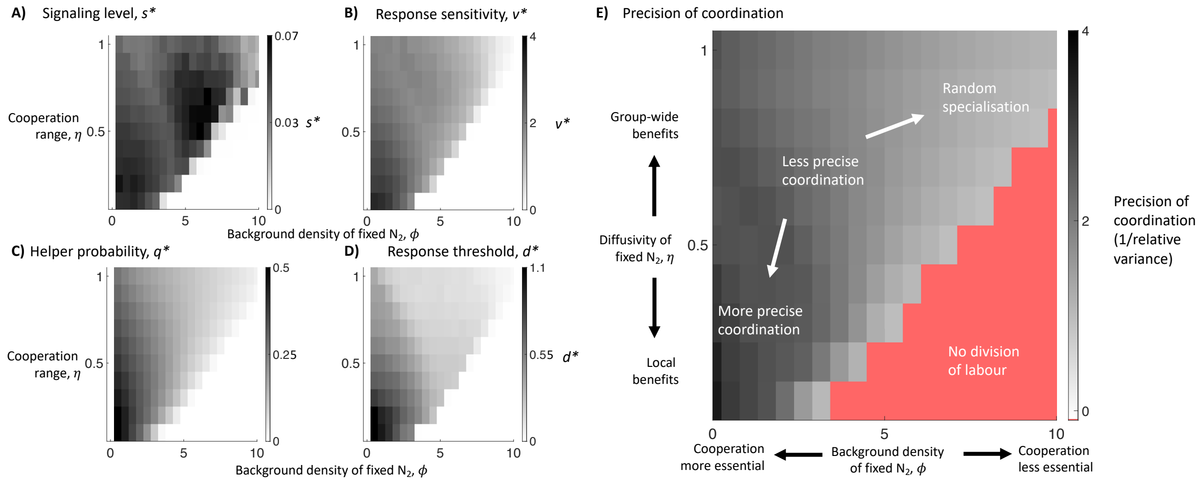

Supplementary Figure 6: **Simulation results for alternate starting conditions (cyanobacteria model)**. We repeated the main simulations analysis while assuming that filament spores consist of 4 cells with no helpers (i.e., a sequence of 4 reproductives: R-R-R-R). We found the same qualitative results as for the main analysis (Figure 5 in the main text): a lower background density of fixed nitrogen (smaller ϕ) and more limited diffusion of fixed nitrogen (smaller η) produced filaments that evolved to a relatively more precise allocation of labour (higher coordination).

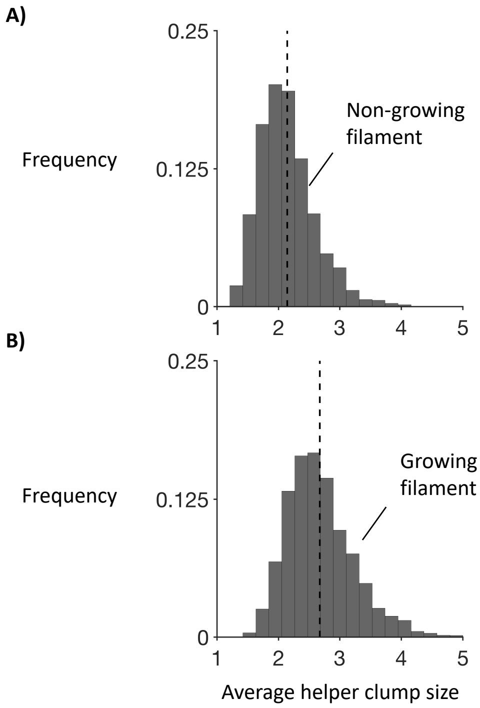

Supplementary Figure 7: **Growing groups produces larger helper clumps (cyanobacteria model)**. We considered the distribution of average helper clump sizes for the cases where: A) helper and reproductive roles are randomly assigned in the final generation of group growth (uncoupling division and differentiation), and B) helper and reproductive roles are assigned as the group grows (division and differentiation coupled). We specifically examined the case where cooperation was essential ($\phi = 0$) and very limited diffusion of fixed nitrogen ($\eta = 0.1$), performing 10,000 independent simulations for each strategy. We found that helper clumps still occur when division and differentiation are uncoupled but that average clump size increases when differentiation occurs as the group grows (average clump size for uncoupled: 2.14 and coupled: 2.69). Source data are provided as a Source Data file.

Supplementary Table

Supplementary Table 1: **Notation and values (cyanobacteria model)**. Evolutionary traits, model parameters, and simulation parameters of the cyanobacteria model, with their definitions and associated values in the presented simulations. Parameters of the evolutionary simulation such as the number of generations per simulation, the mutation rate or size, or the number of independent replicates are not shown here (but see Methods).

**Evolutionary traits**		
**Notation**	**Definition**	**Value(s)**
q	Helper probability in the absence of coordination.	$0 \leq q \leq 1$
s	Relative amount of signal produced by helpers.	$0 \leq s \leq 1$
d	Response threshold to signal by new cells.	$d \geq 0$
v	Response sensitivity to signal by new cells.	$v \geq 0$

**Model parameters**		
**Notation**	**Definition**	**Value(s)**
ϕ	Environmental background density of fixed N_2.	$\phi \in \{0, 0.5, \ldots, 9.5\}$
η	Relative diffusivity of fixed N_2 produced by helpers. Lower values means that benefits are shared more locally.	$\eta \in \{0.1, 0.2, \ldots, 1\}$
$\bar{\phi}$	Maximum rate of N_2 production by helpers.	50
ψ	Maximum growth-rate of reproductives.	10
μ	Shape of reproductive growth-rate as a function of fixed N_2 intake. Higher values means more diminishing.	0.25
$\bar{\pi}$	Critical size at which reproductives replicate (with no response sensitivity.)	100
β	Shape of increase in critical replication size due to higher response sensitivities. Larger values means a more accelerating cost.	0.5
λ	Maximum signal production rate by helpers.	30
ζ	Shape of trade-off between signal production and N_2 fixation.	2.5
ξ	Diffusion range of the signal. Lower values means that the signal decays rapidly along the filament.	0.5
σ_ϵ^2	Noise in local signal detection by new cells.	0.01
L	Size of filament at end of group life-cycle.	50

**Simulation parameters**		
**Notation**	**Definition**	**Values**
σ_q^2	Mutation variance in helper probability, q	1×10^{-3}
σ_s^2	Mutation variance in signal production, s	1×10^{-5}
σ_d^2	Mutation variance in signal response threshold, d	1×10^{-2}
σ_v^2	Mutation variance in signal response sensitivity , v	5×10^{-2}

Supplementary References

[1] Martin Ackermann, Bärbel Stecher, Nikki E Freed, Pascal Songhet, Wolf-Dietrich Hardt, and Michael Doebeli. Self-destructive cooperation mediated by phenotypic noise. *Nature*, 454(7207):987, 2008.

[2] Freddy Bugge Christiansen. On conditions for evolutionary stability for a continuously varying character. *The American Naturalist*, 138(1):37–50, 1991.

[3] Kris De Jaegher. Harsh environments: Multi-player cooperation with excludability and congestion. *Journal of theoretical biology*, 460:18–36, 2019.

[4] Médéric Diard, Victor Garcia, Lisa Maier, Mitja NP Remus-Emsermann, Roland R Regoes, Martin Ackermann, and Wolf-Dietrich Hardt. Stabilization of cooperative virulence by the expression of an avirulent phenotype. *Nature*, 494(7437):353, 2013.

[5] Francisco Dionisio and Isabel Gordo. The tragedy of the commons, the public goods dilemma, and the meaning of rivalry and excludability in evolutionary biology. *Evolutionary Ecology Research*, 8(2):321–332, 2006.

[6] Ilan Eshel, Uzi Motro, and Emilia Sansone. Continuous stability and evolutionary convergence. *Journal of Theoretical Biology*, 185(3):333–343, 1997.

[7] Enrique Flores and Antonia Herrero. Compartmentalized function through cell differentiation in filamentous cyanobacteria. *Nature Reviews Microbiology*, 8(1):39, 2010.

[8] Edgar N Gilbert. Random graphs. *The Annals of Mathematical Statistics*, 30(4):1141–1144, 1959.

[9] Matthew D Herron, Jeremiah D Hackett, Frank O Aylward, and Richard E Michod. Triassic origin and early radiation of multicellular volvocine algae. *Proceedings of the National Academy of Sciences*, 106(9):3254–3258, 2009.

[10] R Fredrik Inglis, Alex R Hall, and Angus Buckling. The role of 'soaking' in spiteful toxin production in pseudomonas aeruginosa. *Biology letters*, 9(1):20120569, 2013.

[11] Vassiliki Koufopanou. The evolution of soma in the volvocales. *The American Naturalist*, 143(5):907–931, 1994.

[12] Valentina Rossetti, Bettina E Schirrmeister, Marco V Bernasconi, and Homayoun C Bagheri. The evolutionary path to terminal differentiation and division of labor in cyanobacteria. *Journal of Theoretical Biology*, 262(1):23–34, 2010.

[13] Lana Strmecki, David M Greene, and Catherine J Pears. Developmental decisions in dictyostelium discoideum. *Developmental biology*, 284(1):25–36, 2005.

[14] Jan-Willem Veening, Oleg A Igoshin, Robyn T Eijlander, Reindert Nijland, Leendert W Hamoen, and Oscar P Kuipers. Transient heterogeneity in extracellular protease production by bacillus subtilis. *Molecular systems biology*, 4(1):184, 2008.
